# The importance of prevention programs to reduce bullying: A comparative study

**DOI:** 10.3389/fpsyg.2022.1066358

**Published:** 2023-01-12

**Authors:** Vanesa Sainz, Beatriz Martín-Moya

**Affiliations:** Faculty of Education and Psychology, Universidad Francisco de Vitoria, Pozuelo de Alarcón, Spain

**Keywords:** bullying, prevention, programs, TEI, KIVA, Mediation Teams

## Abstract

Bullying is a growing and increasingly worrying phenomenon. In recent years, a number of different bullying prevention programs have been implemented to create a more positive school environment. This paper offers a description of these prevention programs, focussing on the three most commonly implemented in schools: TEI, KiVa and Mediation Teams. A qualitative, descriptive and comparative analysis was made using semi-structured interviews with tutors, coordinators and experts at nine schools, three schools for each of the programs. The results show that these bullying prevention programs help raise awareness of this problem within the entire school community, improving the school environment and reducing conflict and instances of bullying. Overall, participating schools reported being highly satisfied with the results of these programs and it is therefore important to consider the mandatory implementation of bullying prevention programs in all schools.

## 1. Introduction

Bullying has been an unfortunate reality since education has been imparted to students grouped in classrooms. Whether referring to ‘mobbing’ or ‘bullying’ (Olweus, 2010), aggression and harassment in schools has received wide attention and great efforts have been made to prevent and eliminate this behaviour. Instances of bullying have become increasingly prevalent, not only because victims are more willing to report situations of abuse but because, unfortunately, the number of cases is simply greater year by year (Medina and Reverte, 2019).

The term ‘mobbing’ was coined by Lorenz (1971) to refer to instances in which a group of animals will attack a single victim. However, in his study of classroom behaviour in Norway during the 1990’s Olweus (2010) used the term ‘bullying’. Attempting to clearly identify this behaviour, Olweus conducted a survey, concluding that ‘bullying’ refers specifically to the exposure of a victim to aggression on repeated occasions occurring over an extended period of time.

To clarify the meaning of the term bullying, it is important to differentiate bullying from other types of aggression and abuse. Authors such as García-Piña and Posadas-Pedraza (2018) conducted a study to define bullying in schools, identifying three key aspects which characterise bullying: it is a phenomenon which occurs within an educational environment repeatedly over time; that is, the abuse is frequent; it is intentional; and the victim suffers feelings of defencelessness, unable to escape from the situation. These three factors are necessary to consider instances of abuse or harassment as “bullying.”

Bullying can be categorised in many ways, given that no instance of bullying is the same. Piñuel and Oñate (2005) consider bullying as not only physical abuse but also psychological and verbal. However, in attempting to identify the different types of school bullying, various authors (Caballo et al., 2011; Smith, 2018) have proposed the existence of five types of bullying: 1. Physical abuse: this type of abuse is characterised by aggressive physical behaviour directed at the victim (pushing, punching, etc.). 2. Relational abuse: characterised as behaviour intended to isolate the individual, damaging their relations with their friends and classmates. 3. Verbal abuse: verbal attacks through insults and humiliations. 4. Cyberbullying: this is a form of harassment that has appeared in recent years along with the development of technologies, taking place on chats, email or social media such as Instagram or Facebook used to insult, humiliate or threaten other people. 5. Carnal abuse: a form of harassment through sexist or chauvinist comments towards the victim or, more seriously, derogatory comments about the body or physical appearance of the victim or intimate touching without their consent.

Within the school context there is an interaction among the three different actors involved. It is important to clearly identify these actors. The first is the victim, the person suffering the aggression, becoming the target of the bully. Victims of bullying are generally insecure or introverted, with low self-esteem and with difficulties socialising (Arias, 2014). The second actor is the aggressor or bully, often characterised as one who uses force as a means of domination. Bullies often have an impulsive character with a tendency to violence, with little empathy or self-control. They often have difficulty in accepting rules, have poorly developed social skills or the ability to resolve conflicts and are often exposed to violence and aggression (Cerezo, 2009). Finally, there are the observers or spectators; those who witness the aggression (Arias, 2014). Spectators can play an active role in abuse, helping the aggressor or the victim; or be passive, merely observing the aggression without taking any action to prevent what they are witnessing. A great deal of research into the phenomenon of bullying has highlighted the important role played by spectators. It has been demonstrated that when these actors take action against the aggression, reporting it or supporting the victim, abusive behaviour diminishes or may even disappear entirely (Conde-Vélez and Ávila-Fernández, 2018).

With this explanation of the characteristics and participants in incidents of bullying we can analyse the evolution of the phenomenon of bullying along with students themselves through different stages of education.

Pre-primary Education is an important stage of development for children, both cognitively and psychologically. A number of studies have found that bullying does not take place during this stage but the signs of aggressive behaviour begin to manifest themselves (Equipo Pedagógico de la Asociación Mundial de Educadores Infantiles, 2018). There has been very little research into the phenomenon of bullying during the earliest years of school. However, some authors, such as Bautista (2020), detect signs of bullying at this stage but with a number of differences from later years. According to the study with children aged 2 and 3, young students are not aware of their aggression and act on their own egocentrism. At the age of 4, aggressive behaviour becomes intentional, consciously aggressive, given their level of cognitive, social and psychological development.

When students enter Primary Education, their cognitive and social development is much higher and they can understand bullying, the harm it can cause and the consequences it may have. Thus, it is in Primary Education where the levels of bullying and aggression are highest (Piñuel and Oñate, 2005).

In Secondary Education, instances of bullying generally diminish, virtually disappearing by the later years although in the majority of schools bullying, harassment and aggression continues to be a reality in the classroom during this stage of education (Martínez et al., 2019).

As we have seen, in all stages of education many students suffer from bullying and harassment, which can cause severe physical, emotional, psychological and relational consequences. For this reason, experts in the field have created a number of programs to be implemented in schools to prevent bullying and to take action when this takes place in the classroom.

The following is an outline, in chronological order, of some of the most important programs established to address the problem of bullying in schools.

First, the *Equipos de Mediación* (Mediation Teams) program was created in the 1960’s to resolve conflicts among students through dialogue and with the help of a mediator, finding solutions to problems and fostering a better relation between the persons in conflict (De la Hoz, 2019). It is important that this mediator be someone external and impartial, who is able to foster communication and dialogue between the persons involved (González, 2015). Authors agree on a series of steps to effectively implement the program: first, the acceptance of the mediator (Martín, 2008); second, the mediator makes initial contact with the persons in conflict; third, each person tells their version of events, analysing the situation with the mediator. The second step is to search for solutions through brainstorming sessions with the participants and, finally, to reach a satisfactory solution to the problem (González, 2015). This program has proven effective in improving the atmosphere in schools and social interactions.

The Olweus program was developed in Norway in 1983 to prevent and reduce bullying, aiming to create a safe and positive school environment (Riese and Urbanski, 2018). The program addresses the problem of bullying on different levels (school, classroom, community, etc.) in order to prevent and reduce instances of bullying in Primary and Secondary schools (Riese and Urbanski, 2018). By restructuring the school environment, the inducements and opportunities for bullying are reduced (Gaffney et al., 2021). As the first step in implementing the program, students complete a survey on bullying and based on the results, schools establish a series of norms that students must follow (Hazelden Foundation, 2007).

The PIKAS program was created in 1989, designed not to prevent bullying but to intervene in existing situations of bullying within schools. The objective is to dissuade the aggressor and to raise the awareness of all those involved about the situation (Pérez, 2010). This program takes a slow, gradual approach in changing the attitudes and behaviour of the aggressors (Cáceres, 2009). For this, a series of interviews are conducted in three distinct stages: (1) the first stage consists of individual interviews with the aggressor, the victim and observers/spectators to try to understand the conflict; (2) the second stage is to have a meeting of the participants to assess the evolution of the conflict; (3) the final stage consists of a group interview of all participants in order to reach a point of resolution and reconciliation. The aim of the interviews is to address the roots of the problem and to resolve the conflict (Pérez, 2010).

The SAVE program (*Proyecto Sevilla Anti-Violencia Escolar*) was a pioneer program in Spain created in 1995 aimed at eliminating and preventing bullying in the classroom through the participation of the entre education community (Cedeño, 2020). The program focusses on educating students about tolerance and diversity, understanding feelings and emotions through curricular projects. SAVE consists of four stages, each with specific actions; one of which, for example, is to create social groups and build friendships for those who are socially isolated, thus reducing the likelihood of suffering from bullying; another is the direct intervention of teachers and tutors in situations of bullying. Through these and other actions the aim is to foster a positive school environment (Ortega and Del Rey, 2001).

The *Proyecto Andalucía Anti-Violencia Escolar* (ANDAVE) was created in 1998 to replace the SAVE program, building on the foundations of another project in Seville focussed on developing interpersonal relations through curricular work and projects. This program focusses on self-perception of the school environment, self-perception as victim and the types, places and characteristics of abuse and of aggressors. There are five lines of action: raising awareness, training of teachers and educators, didactic materials and resources, direct attention for the victim and investigation into instances of abuse (Ortega and Del Rey, 2001).

The *Modelo Construir la Convivencia* project (MCC), working to prevent school bullying and cyberbullying can be considered as a continuation of the SAVE and ANDAVE programs. MCC was designed to create a positive school environment and preventing bullying and cyberbullying in line with these previous programs. It focusses of prevention during Primary and Secondary education, working to resolve conflicts and improving communication. The aim is for students to develop their ability to resolve conflicts before bullying takes place. In cases where prevention is not enough, the program includes an intervention stage in which teachers and families work directly with the aggressor and the victim (Ortega-Ruiz and Córdoba-Alcaide, 2017).

The TEI program (*Tutoría entre Iguales*), created in 2002 by Andrés González Bellido for the prevention of school bullying. According to the author (González-Bellido, 2015), TEI was developed with the aim of raising awareness of the entire education community of the importance and gravity of bullying; the program establishes a reference figure for students (tutors) to help resolve conflicts and create a safe and positive environment at school. The TEI has very clear guidelines for implementation; first, school directors or the school board must approve the implementation of the program; once approved teachers receive training in putting the program into practice. This is followed by initiatives to raise awareness among students of bullying and tutors are assigned. An evaluation is later conducted for the entire school to verify the objectives have been achieved. Finally, a report is created on the program which includes proposals for improvements for the following academic year (Hamodi & Jiménez, 2018).

The ABC program in Ireland was created by Mona O’Moore in 2004 after many years of research in which she observed the high prevalence of bullying in Irish schools. O’Moore worked for several years to create the program which was implemented in a number of Primary schools in 2004 (James and O’Moore, 2008). The program is based on the participation and intervention of all members of the school community (teacher, directors, school staff and families). ABC consists of four phases: teacher training, creation of resources and materials, information for parents, and finally, the implementation of the program itself. To be effective, all members of the school community must be aware of the problem of bullying and be motivated to implement the program, assuming responsibilities and taking part in its execution (Hamodi & Jiménez, 2018).

The AVE program was created by Iñaki Piñuel and Araceli Oñate in 2005, with the aim of eliminating bullying in schools. The CEU School (Colegio CEU, 2019), where the program was implemented, reported that the goal of the program was not only to eliminate bullying but also to measure and prevent violence and aggression within the school. There are six principles for the successful implementation of the AVE program: (1) to create a culture of zero tolerance for bullying, (2) to foment the participation and protagonism of students, (3) to provide schools the tools to anticipate and respond to bullying, (4) to evaluate regularly the level of aggression and violence within the school, (5) to react early and quickly, and (6) to create a ‘risk map’ that includes preventive actions. Levels of aggression and violence within the school are evaluated using the AVE and TEBAE tests, in which students indicate their opinion of each of their fellow students. Based on the responses, a ‘risk map’ that identifies students who may be at risk of bullying. The goal is to anticipate and address potential bullying before it arises (Sánchez-Venteo, 2017).

The KiVa program was created in 2007 by Ari Kaukiainen and Christina Salmivalli in Finland aiming to prevent and reduce bullying in schools (KiVa Antibullying Program, 2007). The program works to raise the awareness of students against bullying from a very early age. The principal objective is to prevent bullying, minimising the negative impact on victims and to create a positive environment in schools. The KiVa program has a clear and specific methodology, indicating the steps to be followed and protocols when instances of bullying are detected in the school. KiVa works through two types of measures: universal and specific. Universal measures are preventive, oriented towards educating students (Herkama et al., 2022). On a regular basis (approximately every 2 weeks) teachers give a class on emotions and values, as well as patterns of abusive behaviour in order to recognise when bullying is taking place. Although students have learned about bullying from the beginning of their school experience, instances of bullying may arise. This is when anti-bullying measures come into play, consisting of specific actions aimed at the participants to stop abusive behaviour, implemented when a case of bullying is detected. According to Mäkelä and Catalán (2018), these measures are implemented by a team of KiVa professionals, tasked with resolving conflicts through interviews with victims, aggressors and classmates. This KiVa team consists of three or more adults working in the school (generally teachers) responsible for intervening in the event of a case of bullying (Johander et al., 2021). The success of both of these measures depends on: the training of teachers, the involvement of the entire school community, prevention, oversight, a structured methodology and easy implementation of the program. The KiVa program also includes an online tool for the initial evaluation of the academic year as well as a constant oversight or monitoring of the changes taking place.

The *Buen Trato* program, created in 2007, focusses particularly on the active participation of adolescents. The authors Sánchez and Blanco (2017) explain that the program is grounded in the effort to improve the school environment by encouraging classmates to help each other through active listening of the worries and fears of their fellow classmates. Teachers and experts provide training to a group of students, giving them the tools to provide guidance and support to other students 2 or 3 years younger than themselves. Once students have received training, they in turn become teachers of their classmates, transmitting what they have learned in dealing with uncomfortable or conflictive social situations. The program works to create a climate of trust and understanding, where young people can positively develop their social relations.

The program *Lucha Contra el Acoso Escolar de la Comunidad de Madrid* was developed in 2016, consisting in the creation of a Unidad del Acoso Escolar as a permanent area within schools. This special unit is dedicated to addressing the issue of bullying through training for teachers, inspectors and school directors, giving them the tools and experience necessary to deal with instances of bullying and aggression. It is also responsible for updating and improving the Action Guide for Bullying provided to all schools as well as conducting awareness campaigns about classroom bullying (Consejería de Educación y Juventud, 2020). This plan also addresses the issue of detection, through the use of the SociEscuela Test, an online test available to all schools, designed to identify students at risk of suffering from bullying and to prevent it before it occurs (EducaMadrid, 2020).

Following on this outline of the principal bullying prevention programs within the Spanish education system, Table 1 presents the most significant characteristics of each program: name, author (s), year, target, description, training, materials, stages and evaluation strategies.

**Table 1:** Comparison of bullying prevention programs.

Name	Author	Year	Target	Description	Training	Materials	Stages	Evaluation strategies
Mediation teams	No author	1960	Teachers and students	Program aiming on conflict resolution through the intervention of a third person (mediator).	No	No	Acceptance, Pre-mediation, ‘Tell me’, Locate conflict, Solutions and Agreement	No
Olweus	Dan Olweus	1983	Teachers and students	Program aiming to improve the school environment by promoting good relation among students.	Yes	No	Class level, school level and community level	Yes
Pikas	Anatol Pikas	1989	Teachers and students	Program seeking to reduce instances of bullying by working with the aggressor.	No	Yes	Individual interviews, follow-up interviews and group meetings	Yes
Save	Government of Andalucía	1995	Teachers and students	Program aiming to prevent bullying through education in values and tolerance.	No	Yes	Democracy, cooperative work, education in values and program for victims	No
Andave	Government of Andalucía	1998	Teachers and students	Program aiming to prevent bullying by improving interpersonal relations.	No	Yes	Positive school environment, victim and types of abuse	No
MCC	Government of Andalucía	2001	Teachers and students	Program aiming to prevent bullying by improving interpersonal relations.	No	Yes	Prevention and direct intervention	No
TEI	Andrés González Bellido	2002/2003	Teachers, students and families	Program in which older students assume the role of tutors for younger students.	Yes	Yes	Raising awareness, approval for implementation, training, pairing, evaluation and reporting	Yes
ABC	Mona O’Moore	2004	Teachers, students and families	Program that works with the aggressor and the victim to resolve conflicts.	Yes	Yes	Training, production of materials, information for parents, program launch	No
AVE	Iñaki Piñuel & Araceli Oñate	2005	Students	Prevention program which uses an evaluation test to determine the probability of suffering from bullying.	Yes	Yes	Interviews, evaluation of positive attitudes	Yes
KiVa	Aro Kaukiainen & Christina Salmivalli	2007	Teachers, students and families	Bullying prevention program using personal interviews to reduce aggression and harassment.	Yes	Yes	Universal measures, Specific measures	Yes
Buen trato	Fundación Anar	2007	Teachers and students	Program working on bullying through student teamworking	Yes	Yes	Creation of guide groups, training of participants	No
Lucha Contra EL Acoso	Ministry of Education, Community of Madrid	2016	Teachers and students	Program to improve measures to prevent bullying and aggression.	Yes	Yes	Creation of a school anti-bullying unit, training, awareness campaign	Yes

Source: the authors.

In exploring the characteristics of bullying prevention programs, considered a valuable tool for schools, this paper will use as a reference the three most commonly implemented programs in Spain: the KiVa program, the TEI program and the Mediation Teams program.

The KiVa, program, before arriving in Spain, was implemented in over 90% of schools in Finland given its proven effectiveness in the prevention and reduction of school bullying (Mäkelä and Catalán, 2018). Since its implementation in Spain, the publishing company Macmillan (2020), responsible for the implementation of this program in Spanish schools, reports that over 100 schools have opted for this program, meaning over 30,000 students are benefiting from the program.

Regarding the TEI program, Saura (2018) reports that over the course of 15 years, this program has been implemented in over one thousand schools in Spain, with over 30,000 trained teachers and over a million students tutors or tutored in the program for the reduction of bullying in their schools.

The Mediation Teams program has a long history in Spain. As noted by Viana-Orta (2018), the program has benefited from over 25 years of experience. Since its first implementation in the Basque Country in the 1990’s the program has spread to the rest of the Autonomous Communities. The program continues to be one of the most widely used program in Spanish schools. It is also the only program included in education legislation of the Autonomous Communities which establish the practice of mediation as a method to resolve conflicts and combat bullying.

The purpose of this research project is to verify the effectiveness and functioning of these programs based on the following question: *What are the most important benefits these bullying prevention programs (KiVa, TEI and Mediation Teams) offer to Spanish schools?*

To answer this question, the study compared the three most representative bullying prevention programs currently implemented in Spanish schools: KiVa, TEI and Mediation Teams (*Equipos de Mediación*). The study also had a number of additional specific objectives:

- To identify the most significant causes or drivers that led schools to implement these types of programs.
- To identify the participants and target of these bullying prevention programs.
- To describe the training provided to each of the agents involved in these programs.
- To observe the evaluation and oversight of these programs.
- To assess the effectiveness and level of school satisfaction with bullying prevention programs.

## 2. Materials and methods

This is a qualitative study using a descriptive-comparative methodology to evaluate the implementation, functioning and effectiveness of various bullying prevention programs implemented in schools. Using a qualitative paradigm, the study compares and contrasts the characteristics and methodologies of these programs and their results in school across the country.

The dependent variable of the study, to evaluate the functioning of the prevention programs, is structured into six categories: the causes of implementation, the target, training received, effectiveness, evaluation and general satisfaction with the program. Additionally, the independent variable is the type of prevention program focussing specifically on three: KiVa, TEI and Mediation Teams.

The sample consisted of nine schools, three for each of the programs being evaluated (three of KiVa, three of TEI and three of Mediation Teams), within the Community of Madrid, represented in the study by the coordinators or directors of the anti-bullying programs. Participating schools were selected using a non-probability sampling method among schools within the Community of Madrid which had implemented one of the three prevention programs subject to this research. The relevant data is provided in Table 2 below.

**Table 2:** Schools participating in the study.

School	Type of program	Implementation period	Type of school
KiVa 1	KiVa	5 years (2016)	Private
KiVa 2	KiVa	4 years (2017)	Charter
KiVa 3	KiVa	5 years (2016)	Private
TEI 1	TEI	2 years (2019)	Charter
TEI 2	TEI	5 years (2016)	Public
TEI 3	TEI	5 years (2016)	Public
Mediation teams 1	Mediation Teams	17 years (2004)	Public
Mediation teams 2	Mediation Teams	12 years (2009)	Charter
Mediation teams 3	Mediation Teams	21 years (2000)	Public

Source: the authors.

The study was based on the results of interviews with the participants. A series of *ad hoc*, semi-structured interviews were conducted using an instrument of 11 questions to gather information about the functioning and effectiveness of the various bullying prevention programs in schools. The interviews were structured into three blocks: The first block focussed on the general characteristics of the program; the second block dealt with the implementation of the program in the school, with questions about the time the program has been in place, reasons for the choice of program, the participants, the training received to implement the program, etc.; the third and final block explored the opinion of the interviewees on the effectiveness of the program, it’s strengths and weaknesses, etc.

The format of the interviews and the measurement instrument were adapted to an online format and implemented by email and by telephone.

The next step was to create a database with the contact details of school which had implemented one of the three bullying prevention programs subject to this paper. This information was collected *via* the internet or through personal contacts. The schools were then contacted by email, requesting their participation and attaching the interview questionnaire for participants to respond online. Participants were also provided with the contact details of the researchers, telephone number and email, to be available to resolve and problems or doubts during the interview process.

The data was analysed using the triangulation technique of the Atlas-ti program, with an exhaustive comparison of the responses from different schools where the programs were implemented. The facilitate analysis, the information was structured into six variables or categories (cause, target, training, evaluation, effectiveness and satisfaction) to organise, compare and contrast the responses of participants.

## 3. Results

In evaluating the results of the interviews, the responses of tutors and administrators regarding the prevention programs in different schools were compared and analysed according to the research variables. The results were structured according to the six variables being evaluated: cause, target, training, program evaluation, effectiveness and school satisfaction.

### 3.1. Causes

This section offers an analysis of the reasons why school chose one the bullying prevention programs KiVa, TEI or Mediation Teams. While all of these programs are aimed at preventing bullying in schools, not all the schools chose these programs for this reason. One KiVa school and another Mediation Teams school reported implementing these programs to deal with growing conflicts between students that was leading to instances of bullying. This was confirmed by the school Mediation Teams 1: *“because the school environment is much more complex than before,”* while school KiVa 2 noted that *“three years ago we began to have lots of problems and conflicts in the higher grades*.” However, the rest of the schools implementing the KiVa, TEI or Mediation Teams programs did so not because of a need for intervention but to *“to improve the school environment*” as the Mediation Teams 2 school reported. The coordinator of the Mediation Teams 3 school added that *“it is an effective way to deal with conflicts and to improve the school environment*.” Many school administrators similarly reported their concern about the ignorance about the issue of bullying in general. This is another reason cited by schools for their decision to implement one of these programs; that is, that the program would serve, not only to improve the atmosphere in the school but also to raise awareness among school community of the issue of bullying. This is reflected in the observation by school TEI 3 *“that the school has no serious conflict problems but there is concern about the ignorance of the gravity of school bullying*”; KiVa 3 school affirmed that “*it is socially imperative to eliminate bullying and to make it a real priority,”* while the Mediation Teams 3 school noted that through these programs *“students learn important values, such as dialogue and communication to resolve conflicts.”*

### 3.2. Target

Another aspect analysed in the study were the target. To whom the program is aimed and who works with it. All programs aim to involve the entire school community in order to improve the culture of the school and create a safe and positive environment for learning. The coordinator of the school Mediation Teams 2 reports that *“the entire school community is involved.”* In all programs the principal actors are the students. For example, school TEI 3 noted that *“the program is followed by the whole school community, but the main protagonists are the students.”* Teachers also work with the students, either directly or indirectly, providing guidance and support where and when necessary. For example, the coordinator of the school TEI 3 affirmed that “*the teachers help students develop their conflict resolution skills.”* In no cases did families participate in the start-up of the program. However, as indicated by school KiVa 3 *“parents are informed about the measures implemented through the program*.” The TEI schools also held a training session for families. Thus, the three programs share the same target, the protagonists are the students, while the rest of the school community is available at all times to help carry out the programs.

### 3.3. Training

The third aspect is training, that is, the type of instruction on the programs received by teachers and school professionals. The schools which implement KiVa and TEI receive specialised training about the programs through courses imparted by experts in the implementation and application of these programs. The tutor at the school KiVa 2, reported that at their school *“all teachers received a training course of 10 to 15 h in three sessions.”* The coordinator at school KiVa 1 added that *“the publisher McMillan provided the training for all teachers.”* In the case of the TEI program, school TEI 3 explained that *“two 4-h sessions of presential training are provided, plus a one-hour remote training session.”* School TEI 2 noted that *“both the presential and online sessions were given by a TEI trainer.”* For the Mediation Teams program training is not regulated and diverse types of training is provided depending on the aspects schools wish to focus on. Thus, each school with Mediation Teams receives a different type of training. The coordinator of the school Mediation Teams 3 explained that “*the teachers train themselves through seminars or with the help of the local authorities”* and the coordinator of the school Mediation Teams 2 reported that there was *“a course by an expert in school relations from the University of Alcalá.”*

### 3.4. Evaluation

The surveys and evaluations of the programs offer a general overview of how they work and the level of school satisfaction. As mentioned above, the KiVa and TEI programs are highly structured; both programs include evaluation surveys to measure the effectiveness of the program. According to school KiVa 3, these surveys “*are made by the students at the beginning and end of the year.”* The evaluation of the TEI program, according to the school TEI 3 *“consists of a survey made by the students and teachers every quarter on levels of satisfaction, feelings of wellbeing, type of relations,* etc.*”* The majority of school gather this data not only to assess the functioning of the program but also to propose changes and *“proposals for improvements,”* according to school TEI 3. Compared to KiVa and TEI, the Mediation Teams program is less structured or staged, with no established evaluation system in the program; in these cases the schools themselves organise their own surveys. The school Mediation Teams 1 reported that “*se the program is done at the end of the year with the participation of teachers, students and families*.” At school Mediation Teams 3, rather than use the surveys, they *“evaluate the conflicts that arose during the year to see if these were resolved*.” It should be noted that all evaluations of all programs by all schools were very positive.

### 3.5. Effectiveness

By analysing this variable, the aim is to determine to what extent bullying is truly being reduced and classroom environments improved. Thus, it can be observed that all schools reported an improvement in the classroom environment. As the school KiVa 2 responded *“there have been fewer conflicts and the students are more aware of the problem.”* School TEI 3 also reported *“notable changes, principally among tutors, who feel themselves the protagonists of the positive environment in the school. Incidents of conflicts have notably decreased.”* Furthermore, as noted by the school Mediation Teams 2, *“the atmosphere is different from before; there is more help between classmates and the awareness of a good environment is real among the students*,” while school KiVa 1 reported that the program *“spurs the students to actively participate, enabling them to intervene to manage minor conflicts*.”

### 3.6. Satisfaction

This final variable aims to evaluate the level of satisfaction of schools with the program. Experts in KiVa affirm *“they are very pleased and that the program meets their objectives”* (KiVa 2). Schools with the TEI also reported *“a more positive atmosphere in the school can be perceived”* (TEI 2). Schools implementing the Mediation Teams program also reported their satisfaction: according to the Mediation Teams School 3 *“there is a good atmosphere and relations among students.”*

Although the level of general satisfaction is high, schools of the KiVa and TEI programs propose certain improvements to adapt these programs to their specific needs. In both cases, school propose improvements in program materials, with school TEI 3 suggesting *“better technological or digital materials,”* and school KiVa 1 recommending *“better adaptation of the materials to the ages of the students.”*

Thus, it can be concluded that, in general terms, all schools are pleased and satisfied with their anti-bullying programs, which are effective in preventing and reducing instances of aggression and bullying in the classroom.

Table 3 below offers a structure comparison of the bullying prevention programs KiVa, TEI and Mediation Teams by causes, target, training, evaluation, effectiveness and satisfaction with the programs.

**Table 3:** Results of the KiVa, TEI and mediation teams programs.

Name	KiVa	TEI	Mediation teams
Causes	Prevention of bullying. Awareness campaign about bullying. Conflict resolution.	Manage a positive school environment. Raise awareness about bullying. Prevention of bullying.	Eliminate conflicts. Improve school environment. Educate values.
Target	Students. Tutors, teacher also participate.	Students. The entire school community. Training of teachers, students and families.	Students. The entire school community.
Training	Specialised presential and online courses. Additional program materials.	Specialised presential and online course. Offers materials.	No formal, structured training. Training at universities or local councils.
Evaluation	Survey of students evaluating the effectiveness of the program.	Questionnaires for teachers and students each semester to evaluate the effectiveness of the program.	Subjective evaluation using own method. Study of cases or surveys.
Effectiveness	Improved relations in schools. Reduction of instances of aggression and bullying. Greater awareness of bullying.	Improved relations in schools. Greater awareness of bullying. Reduction of conflicts.	Effective program. Improves the environment in schools. Reduction of conflicts.
Satisfaction	Positive opinion of the program. Schools are satisfied.	Very positive opinion of the program. Schools are satisfied.	Good levels of satisfaction on the part of schools.

Source: the authors.

## 4. Discussion

The responses from school administrators and program directors from a number of schools offer an overview of the experiences and opinions regarding these prevention programs. Firstly, the study found that, while each are very different, the KiVa, TEI and Mediation Teams programs were all highly rated by schools which generally agreed that the programs meet the needs of the school and are effective in preventing and stopping instances of aggression and bullying in the classroom. These responses indicate that the three programs are truly effective in preventing and eliminating instances of bullying and aggression in schools, the principal aim of these programs.

Schools implemented these anti-bullying programs to create a more positive school environment, reduce conflicts and to prevent bullying. The reasons cited by schools are all very similar regardless of the specific program, fully agreeing with the general intentions and purposes underlying these programs. Some program coordinators go further, affirming their belief that addressing the issue of bullying is a pressing social need and of the importance of educating students in positive values and communication, giving them the skills to resolve conflicts.

The three programs all aim to involve the entire school community with students always playing a central role, in line with Cross et al. (2021). Only the TEI program includes a structured training plan for families although some school using Mediation Teams intend to involve parents more directly in the coming academic years.

Training is also an important aspect of bullying prevention programs. At TEI and KiVa schools this training is highly structured and is the same for all schools implementing these programs, where teachers receive between 10 and 20 h of training divided into two or three sessions and which also include resources and materials for both teachers and students and, in the case of TEI, for families as well. The Mediation Teams program is less structured with diverse forms of training.

Professionals and coordinators of the programs all report an improvement in the school environment and a reduction of conflicts since their implementation. Furthermore, the study shows that students are also more aware and vigilant about the problem of bullying, feeling themselves the protagonists and active participants in preventing instances of violence and creating a positive atmosphere in their school.

To verify the functioning and effectiveness of bullying prevention programs, TEI and KiVa, being the most highly structured, include an evaluation survey. The KiVaa survey, conducted at the start and the end of the academic year, is aimed exclusively at students. The TEI surveys are completed by both students and teachers every semester. The Mediation Teams program does not include a formal evaluation mechanism and the centres themselves decide on their methods of evaluation.

As noted by Avilés et al. (2008), the Mediation Teams program aims to resolve conflicts through dialogue, communication and the participation of a mediator. The results of our research show that this program is effective according to reporting from the schools themselves. Although no studies have been conducted which clearly show a reduction in cases of bullying (Usó et al., 2009), schools report they have noted a difference in classrooms where there is a more positive environment with fewer conflicts.

The TEI program for the prevention of bullying was created to raise awareness of this issue among the entire education community (González-Bellido, 2015). A number of schools have implemented this program, not only to eliminate classroom conflicts but also, as the program itself explains, to heighten awareness of the reality and the gravity of bullying. Studies of this program (González-Bellido, 2015) show it to be very effective, reducing instances of bullying and aggression in schools to zero. Given this success, many schools have implemented this program, all reporting it to be highly effective.

The KiVa program to prevent and eliminate bullying in schools (KiVa Antibullying Program, 2007) has been highly effective in the schools where it has been implemented, reducing instances of conflict. According to available figures, the program has successfully reduced bullying in schools by some 80% (KiVa Antibullying Program, 2007). The results of our study confirm a significant reduction in cases of bullying and conflict.

Schools reported being highly satisfied with their bullying prevention initiatives, regardless of the specific program implemented. Schools using more structured and guided programs (TEI and KiVa) agree that part of this success is due to the structured nature of the program, allowing step by step implementation. However, schools using the Mediation Teams program reported feeling lost when beginning the implementation process given the less structured nature of the program. Despite their satisfaction, the TEI and KiVa program coordinators have identified areas for improvement, particularly noting that materials should be more closely adapted to the age of the students, their educational circumstances, and social realities.

## 5. Conclusion

The present study offers an in-depth look at some of the anti-bullying programs currently being implemented in Spain, through an analysis of the responses and opinions of experts and coordinators of these programs.

The coordinators of these programs report an improvement in the school environment and better relations both among students and between students and teachers since the implementation. These changes clearly demonstrate that initiatives to prevent bullying have a positive impact on the school environment regardless of the specific program. Thus, it is essential to heighten public awareness about the importance of this issue and the need to address and eliminate harmful and abusive behaviour in schools.

The general assessment of all three bullying prevention programs (KiVa, TEI and Mediation Teams) is very positive. Experts agree that these programs meet the needs of the school communities and are effective in preventing, identifying and stopping instances of bullying. It has been seen that, prior to the implementation of these programs, the issue of bullying was silenced, almost a taboo subject, about which most people preferred not to speak. The programs have served to heighten awareness of the importance and gravity of bullying throughout the school community (students, teachers and families) generating a culture of zero tolerance for violence. The result has been a decrease in conflicts and instances of bullying and abusive behaviour in all of the participating schools.

Given the circumstances of the COVID-19 pandemic, the limitations on this study have been considerable. The number of participants was to be much higher initially and interviews were to have been conducted in person. However, although the sample size was not very large, the responses of schools were very similar and it may be supposed that a larger sample would not have provided any relevant additional data. Sufficient information for the purposes of this study was obtained using a limited sample although it should not be considered as representative.

Analysing the results obtained in the study, it would be instructive to explore further the different anti-bullying programs implemented throughout Spain, with the participation of more schools representative of the entire country, in order to conduct a comparison on a national scale. Furthermore, it would be helpful to expand the sample to include other types of prevention programs in order to observe first-hand the differences and benefits of the various programs implemented in schools nationwide.

It is important to underscore the important role of governments and public authorities in the prevention of a problem which causes daily harm to girls and boys in schools (Cantera et al., 2021). Public institutions must provide schools with the funding and resources necessary to undertake these initiatives. Furthermore, it is important that governments enact legislation to support the actions of schools to create a safe learning environment and protect students from violence and abuse.

It would also be instructive to know the measures undertaken in other countries in this area. A comparative study of the legislation and protocols established across Europe would help identify the measures which are most effective in preventing bullying in schools.

Given the conclusions of this research project, one may imagine a future where these types of programs are universal and obligatory for all schools with the support of government authorities and applicable legislation. Universal application of prevention programs would enhance the classroom environment, raise awareness of the issue of bullying among the entire education community, creating a safe and positive environment for students where they can happily learn, grow and reach their full potential.

## Data availability statement

The raw data supporting the conclusions of this article will be made available by the authors, without undue reservation.

## Ethics statement

Ethical review and approval were not required for the study on human participants in accordance with the local legislation and institutional requirements. Written informed consent from the [patients/ participants OR patients/participants legal guardian/next of kin] was not required to participate in this study in accordance with the national legislation and the institutional requirements.

## Author contributions

VS and BM-M equally contributed and wrote the manuscript. All authors contributed to the article and approved the submitted version.

## Funding

This research was conducted through a project of the Call for Research Grants of the Universidad Francisco de Vitoria (Ref.: UFV2021-09).

## Conflict of interest

The authors declare that the research was conducted in the absence of any commercial or financial relationships that could be construed as a potential conflict of interest.

## Publisher’s note

All claims expressed in this article are solely those of the authors and do not necessarily represent those of their affiliated organizations, or those of the publisher, the editors and the reviewers. Any product that may be evaluated in this article, or claim that may be made by its manufacturer, is not guaranteed or endorsed by the publisher.

## References

[ref1] Arias W. (2014). ¿Qué es el bullying?: Los actores, las causas y los principios Para su intervención [what is bullying?: the participants, causes and principles to its intervention]. Revista de psicología de Arequipa 4, 11–32.

[ref2] Avilés J. Torres N. Vian M. (2008). Equipos de Ayuda, Maltrato entre Iguales y Convivencia Escolar [Help Teams, Peer Abuse and School Coexistence]. Electron. J. Res. Educ. Psychol. 6, 863–886.

[ref3] Bautista B. (2020). Afrontando el acoso escolar desde Educación Infantil [facing bullying from early childhood education]. Revista de Innovación Didáctica de Madrid 64, 70–84.

[ref4] Caballo V. Arias B. Calderero M. Salazar I. Irurtia M. (2011). Acoso escolar y ansiedad en los niños (I): Análisis de su relación y desarrollo de nuevos instrumentos de evaluación [School bullying and social anxiety in children (I): analysis of your relationship and development of new evaluation instruments]. Behavioral Psychol. 19, 591–609.

[ref5] Cáceres A. (2009). An alternative from awareness and reflection for the effective development of interpersonal relationships and the reduction of school bullying among peers [Bachelor’s thesis]. Universidad de la Sabana.

[ref6] Cantera L. Váquez M. Pérez A. (2021). Bullying situation in Spain: laws, prevention and care. Revista Olhares 9, 05–20. doi: 10.34024/olhares.2021.v9.10622

[ref7] Cedeño W. (2020). La violencia escolar a través de un recorrido teórico por los diversos programas Para su prevención a nivel mundial y latinoamericano [school violence through a theoretical tour of the various programs for its prevention worldwide and in Latin America]. Revista Universidad y Sociedad 12, 470–478.

[ref8] Cerezo F. (2009). Analysing bullying in Spanish schools. Int. J. Psychological Therapy 9, 367–378.

[ref9] Colegio CEU. (2019). Programa AVE: la prevención es la mejor herramienta contra el acoso escolar. Blog Colegio CEU San Pablo Montepríncipe. Available at http://www.colegioceumonteprincipe.es/blog2/programa-ave-contra-el-acoso-escolar-la-prevencion-es-la-mejor-herramienta/

[ref10] Conde-Vélez S. Ávila-Fernández J. A. (2018). Influence of bystanders on aggression and a sense of school bullying. Psychol. Soc. Educ. 10, 173–187.

[ref11] Consejería de Educación y Juventud. (2020). Programa Lucha contra el Acoso escolar [program fight against school bullying]. Available at https://www.comunidad.madrid/transparencia/informacion-institucional/planes-programas/programa-lucha-acoso-escolar

[ref12] Cross D. Runions K. Pearce N. (2021). Friendly schools’ bullying prevention research: implications for school counsellors. J. Psychol. Couns. Sch. 31, 146–158. doi: 10.1017/jgc.2021.19

[ref13] De la Hoz C. (2019). Mediación escolar: un proceso de resolución de conflictos y prevención de bullying en centros educativos [School mediation: a conflict resolution process and bullying prevention in educational centers]. Revista Familia 57, 177–186.

[ref14] EducaMadrid. (2020). Programa de Lucha contra el Acoso Escolar. Comunidad de Madrid. Available at https://www.educa2.madrid.org/web/convivencia/-quees-sociescuela-/-/visor/programa-de-lucha-contra-el-acoso-escolar-comunidadde-madrid

[ref15] Equipo Pedagógico de la Asociación Mundial de Educadores Infantiles. (2018). El bulllying se gesta en la etapa de Educación Infantil. Biblioteca AMEI-WAECE.

[ref16] Gaffney H. Ttofi M. M. Farrington D. P. (2021). Effectiveness of school-based programs to reduce bullying perpetration and victimization: an updated systematic review and meta-analysis. Campbell Syst. Rev. 17:e1143. doi: 10.1002/cl2.1143 PMC8356322 37131921

[ref17] García-Piña C. Posadas-Pedraza S. (2018). Acoso escolar: de lo tradicional a un enfoque integral [bullying from traditional to an integrated approach]. Acta Pediátrica de México 39, 190–201. doi: 10.18233/APM39No2pp190-2011579

[ref18] González I. (2015). Mediation in cases of school bullying [Bachelor’s thesis]. Universidad de Valladolid.

[ref19] González-Bellido A. (2015). Programa TEI “Tutoría entre Iguales” [TEI program “peer tutoring”]. Innovación educativa 25, 17–32. doi: 10.15304/ie.25.2854

[ref20] Hamodi C. Jiménez L. (2018). Prevention models of bullying: What can be done in early childhood education? Revista de investigación educativa de la Rediech 9, 29–50.

[ref21] Hazelden Foundation. (2007). Olweus bullying prevention program. Recuperado de http://www.stgilesschool.org/wp-content/uploads/2015/03/Olweus-Parent-Pamphlet-in-Spanish.pdf

[ref22] Herkama S. Kontio M. Sainio M. Turunen T. Poskiparta E. Salmivalli C. (2022). Facilitators and barriers to the sustainability of a school-based bullying prevention program. Prev. Sci. 23, 954–968. doi: 10.1007/s11121-022-01368-2, PMID: 35467235 PMC9343300

[ref23] James S. O’Moore M. (2008). The effectiveness of a Nationwide intervention Programme to prevent and counter school bullying in Ireland. Int. J. Psychol. Psychol. Ther. 8, 1–12.

[ref24] Johander E. Turunen T. Garandeau C. F. Salmivalli C. (2021). Different approaches to address bullying in KiVa schools: adherence to guidelines, strategies implemented, and outcomes obtained. Prev. Sci. 22, 299–310. doi: 10.1007/s11121-020-01178-4, PMID: 33098542 PMC8032636

[ref25] KiVa Antibullying Program. (2007). Say no to bullying with KiVa! Available at https://espanaes.kivaprogram.net/

[ref100] Lorenz K. (1971). Sobre la agresión: el pretendido mal [About the aggression: the alleged evil]. Madrid: Siglo XXI.

[ref26] Mäkelä T. Catalán B. (2018). Programa de convivencia y anti-acoso escolar KiVa: Impacto y reflexión [KiVa coexistence and anti-bullying program: impact and reflection]. Anales de la Fundación Canis Majoris 2, 234–358.

[ref200] Macmillan. (2020). Solutions for Excellence: Kiva. Available at: https://www.macmillaneducation.es/solutions-for-excellence/kiva/#mas_de_100_coles_kiva

[ref27] Martín G. (2008). La Mediación como Herramienta de Prevención de la Violencia Escolar [Mediation as a Tool for the Prevention of School Violence]. Revista de Mediación 1, 26–31.

[ref28] Martínez I. Gómez E. Goig R. (2019). El acoso escolar en educación secundaria: prevalencia y abordaje a través de un estudio de caso [Bullying in secondary education: prevalence and approach through a case study]. Comunitaria: Revista Internacional de trabajo social y ciencias sociales 17, 71–91. doi: 10.5944/comunitania.17.4

[ref29] Medina J. Reverte M. (2019). Violencia escolar, rasgos de prevalencia en la victimización individual y grupal en la Educación Obligatoria en España [school violence, traits of prevalence in individual and group victimization in compulsory education in Spain]. Revista de estudios y experiencias en educación 18, 97–110. doi: 10.21703/rexe.20191837medina9

[ref30] Olweus D. (2010). Handbook of bullying in schools. An International Perspective. London. Routledge.

[ref31] Ortega R. Del Rey R. (2001). Andalucía antiviolencia escolar (ANDAVE). Revista de Educación. Available at https://idus.us.es/bitstream/handle/11441/87980/20_Convivencia_escolar_un_enfoque_practico-116-139.pdf?sequence=1yisAllowed=y

[ref32] Ortega-Ruiz R. Córdoba-Alcaide F. (2017). El Modelo construir la convivencia Para prevenir el acoso y el ciberacoso escolar [the building school convivencia model to prevent bullying and cyberbullying]. Innovación educativa 27, 19–32. doi: 10.15304/ie.27.4287

[ref33] Pérez V. (2010). Fenómeno bullying aplicación y efectividad del método Pikas en alumnos de cuarto a sexto de Primaria. (Trabajo de Fin de Grado). Universidad de San Carlos, Guatemala.

[ref34] Piñuel I. Oñate A. (2005). Informe Cisneros VII “Violencia y Acoso escolar” en alumnos de primaria, secundaria y bachiller [Cisneros report VII "violence and bullying" in primary, secondary and high school students]. Instituto de Innovación Educativa y Desarrollo Directivo.

[ref35] Riese J. Urbanski J. (2018). Programa Olweus para Prevenir el Acoso Escolar. Biblioteca Jurídica Virtual de la Universidad Nacional Autónoma de México. Available at https://archivos.juridicas.unam.mx/www/bjv/libros/12/5612/5.pdf

[ref36] Sánchez G. Blanco J. (2017). El “Buentrato” programa de prevención del acoso escolar, otros tipos de violencia y dificultades de relación. Una experiencia de éxito con alumnos, profesores y familias [the "Buentrato" program for the prevention of bullying, other types of violence and relationship difficulties. A successful experience with students, teachers and families]. Revista de Estudios de Juventud 115, 115–136.

[ref37] Sánchez-Venteo E. (2017). El bullying y la violencia escolar [the bullying and the school violence]. Revista Internacional de apoyo a la Inclusión, Logopedia, Sociedad y Multiculturalidad 3, 91–105.

[ref38] Saura V. (2018). Tutoría entre iguales: 15 years, mil escuelas y un millón de alumnos [peer tutoring: 15 years, a thousand schools and a million students]. El Diario de la Educación. Sourced on May 6, 2020, from https://eldiariodelaeducacion.com/2018/02/02/tutoria-iguales-15-anos-mil-escuelas-millon-alumnos/

[ref39] Smith P. K. (2018). Commentary: types of bullying, types of intervention: reflections on Arseneault. J. Child Psychol. Psychiatry 59, 422–423. doi: 10.1111/jcpp.12897, PMID: 29574738

[ref40] Usó I. Adrián J. Villanueva M. (2009). La convivencia en las aulas de Secundaria: Programas Alumno Ayudante y Alumno Mediador [Coexistence in Secondary Classrooms: Student Helper and Student Mediator Programs]. Universitat Jaume I, España.

[ref41] Viana-Orta M. (2018). 25 years de mediación escolar en España: 1994-2019. Una cronología de su llegada. Cuestiones pedagógicas 27, 11–22. doi: 10.12795/CP.2018.i27.01

